# The experiences of endometriosis patients with diagnosis and treatment in New Zealand

**DOI:** 10.3389/fgwh.2022.991045

**Published:** 2022-08-31

**Authors:** Katherine Ellis, Deborah Munro, Rachael Wood

**Affiliations:** ^1^Department of Mechanical Engineering, University of Canterbury, Christchurch, New Zealand; ^2^The Biomolecular Interaction Centre, University of Canterbury, Christchurch, New Zealand; ^3^Department of Chemical and Process Engineering, University of Canterbury, Christchurch, New Zealand

**Keywords:** endometriosis, chronic pain, treatment, laparoscopy, hysterectomy, ultrasound, pain relief, diagnosis

## Abstract

Endometriosis is a chronically painful, invasive, inflammatory disease, with limited treatment options and long delays to diagnosis, which impacts 10% of females in New Zealand.

**Introduction:**

As part of a larger group discussion study, this paper covers three themes associated with endometriosis patient experiences: intensity of pain, diagnostic tool shortcomings and perspectives of treatment options.

**Materials and methods:**

The goal of this research was the inclusion of patient voices to guide research priorities. In early 2022, 50 New Zealand endometriosis patients participated in anonymous, asynchronous, text-based group discussions on the VisionsLive platform. The patients ranged in age from 18–48. The patients answered 50 questions, 23 text-based and 27 quantitative, and then took part in online group discussions.

**Results and discussion:**

The average age of symptom onset was 15.3 years, while the average delay from symptom onset to a working or surgically confirmed diagnosis was 7.91 years. The top five reported symptoms within the cohort were pain-based, and the participants discussed the many impacts of this pain on their work and education. The four main diagnostic tools employed on this cohort were abdominal ultrasound (72%), transvaginal ultrasound (68%), laparoscopy (82%) and sharing their symptom history with a medical practitioner (88%). The most common emotions patients experienced following receiving a diagnosis of endometriosis were relief (86%), feeling overwhelmed (54%), and anger (32%). The main treatments offered to this cohort were pain relief (96%), laparoscopic surgery (84%) and the combined oral contraceptive pill (80%). Of these three treatments, only laparoscopic surgery was viewed positively by the majority of users, with 67% considering laparoscopy an effective treatment, compared to 46% of users for pain relief, and 25% of users for the combined oral contraceptive pill.

**Conclusions:**

Gathering the voice of patients revealed that long delays to diagnosis and dismissal by medical practitioners frequently manifests as a reaction of relief by patients once diagnosed. Results also showed treatment options such as pain relief and hormonal medications were often considered ineffective, but were routinely offered as the first, or only, options for patients. It is therefore important that both quicker routes to diagnosis and more effective treatment options be developed.

## Introduction

Endometriosis is a disease characterized by the presence of endometrial glands and stroma outside of the uterus (1, 2). Endometriosis is an inflammatory condition (3) and has local effects at the positions of ectopic endometrium (lesions outside of the uterus) and broad effects from central sensitization (4). Endometriosis is damaging to the individual, society, and the economy (2), with the medical therapies that are available acting to suppress, not cure, the condition (5). Endometriosis patients exhibit a wide range of symptoms, with a non-exhaustive list including chronic pelvic pain, dysmenorrhea (menstrual pain), deep dyspareunia (pain with sex), dysuria (pain with urination), dyschezia (pain with defecation), mid-cycle pain and metrorrhagia (mid-cycle bleeding), constipation, diarrhea, cramping, infertility, and myofascial pain (6–8).

The experience of pain for endometriosis patients is multifaceted and harms all aspects of quality of life (9). One key impact of endometriosis pain is its chronic nature. Due to the extended period of exposure to pain signals in endometriosis, the body is prone to reclassifying this pain as threatening, altering the normal modulation of this pain (10). During central sensitization, signals of pain are abnormally processed resulting in experiences of pain becoming heightened and exaggerated (10–12). Simultaneously, peripheral sensitization results from the repetitive and prolonged stimulation of a patient's nociceptors, as occurs in endometriosis, progressively lowering the threshold for activation (10, 12). Women with chronic pelvic pain, with or without a surgically-confirmed diagnosis of endometriosis, show significantly lower pain tolerances than healthy controls (4). The effect of suffering from central and peripheral sensitization for endometriosis patients means their pain symptoms often increase over time, even if the disease itself does not appear to progress. The manifestation of endometriosis pain is complex, and the mechanisms that underly the expression and progression of pain symptoms have not yet been fully elucidated (10).

Diagnosis of endometriosis is frequently delayed, placing a substantial burden on patients and those that care about them, as this delay can prevent appropriate clinical management (13). The biggest reason for the delay in diagnosis is that a definitive diagnosis of endometriosis is available only *via* surgery and histological examination of excised tissue (14). Surgery is expensive, onerous for many women to access and traumatic to the body. This last concern is particularly substantial as it may prompt further nervous system trauma and may even initiate further endometriosis development (15).

Treatment options for endometriosis patients are limited. Surgery is the critical diagnostic tool and is considered the gold standard for treatment by removing endometriotic lesions, endometriomas, and deep infiltrating endometriosis (5). Patients with endometriosis can also undergo hormonal treatments to suppress their symptoms (16) or utilize pain relief medications. All three of these treatment options are suboptimal. Surgery is difficult to access, and hormonal treatment methods have many side effects, including mood swings, weight gain and nausea (17). Pain management utilizing common drugs, such as non-steroidal anti-inflammatory drugs (NSAIDs), is not sustainable long-term due to both its health consequences and that it does not suppress or reduce the presence of endometriotic lesions. For the treatment of cancer patients, multi-disciplinary teams have shown improvements to patient outcomes by holistically assessing the status of the patient (18). For endometriosis, holistic pain management with multi-disciplinary teams have included practitioners such as pain specialists, physiotherapists, dieticians and psychologists to produce integrated plans to approach the patient's treatment (19). These types of approaches to pain management have shown to be an improvement to solely using pharmacological medications for pain management (19), however, both cost and time can be prohibitive factors for these types of schemes (18).

Within this article, aspects of a study conducted with endometriosis patients in New Zealand in 2022 are presented. This study covered nine key themes: 1) the intensity of endometriosis pain, 2) experiences of diagnostic tool shortcomings, 3) patient perspectives of treatment efficacies, 4) the effect of patient's lack of knowledge on their experience, 5) the influence of doubt on diagnostic delay, 6) the impact of socioeconomic position on patient capacity to access care, 7) the necessity for more subsidized care, 8) the need for greater research funding and 9) patient desire for improved education and readily available information about endometriosis. This paper represents the quantitative and qualitative findings associated with the first three themes.

## Materials and methods

The present study is part of a larger investigation into the application of biomedical engineering principles for understanding how the human biological system enables endometriotic lesions to become established and grow. Since both diagnostic and treatment options are limited, as are the time and resources to research alternatives, the authors wanted to better comprehend the experiences, perspectives and research priorities of endometriosis patients within New Zealand. It is the patients who face the delays and the hardships, so it should be their voices that inform the most significant needs that must be addressed in their care. The authors hypothesized that collecting this information and knowing more about the stories of endometriosis patients would enhance their future research by making it patient-centered. The collection of this data is also in alignment with the 2017 research goals by the Global Consortium of Endometriosis Investigators for patient perspectives: “patient views on the most pressing topics in endometriosis research and clinical priorities should be sought and subcategorized into different demographic groups, including age, symptoms, ethnicity, and economic background” (20).

### Study design and data collection

In February and March 2022, social media was utilized to advertise for participants for this study, with the invitation shared by the authors, and affiliated organizations, with the invitation then further disseminated independently by members of the public. Endometriosis patients were recruited to participate in an anonymous, online discussion about their experiences with diagnoses and treatment of endometriosis in New Zealand, with fifty endometriosis patients completing the entire discussion. All participants were over the age of 18, resided in New Zealand and had a diagnosis of endometriosis that was either confirmed by surgery or suspected by their GP or OBGYN. After submitting an expression of interest through email or by filling in an interest form on social media, participants were provided with an information sheet detailing the intent and method of the study. Once their interest to participate was confirmed, each participant filled in a physical or online consent form and returned it to the lead researcher before the discussions commenced. No further selection criteria were applied and all participants who expressed their interest, fulfilled the criteria, and filled in the consent form were eligible to participate.

These discussions were asynchronous and ran for at least 72 h. Each participant had a unique link to the discussion and could log in at whatever times suited them, as many times as they wanted to answer questions, read the anonymous responses of others and write replies. To reduce the phenomenon of “groupthink” where individuals accept a perceived group consensus (21), each participant could only see the responses of others once they had submitted their answer to a question. Participants who did not have enough time to complete all of the questions online were given the option to complete the rest of their answers offline and return these to the authors. There were a further three participants who started the discussion but decided not to finish all of the questions. The data from these three participants was removed from the final data set. Furthermore, there were six endometriosis patients who completed the consent form, but did not participate further.

The discussion comprised 50 questions, of which 23 had text answers, and 27 were quantitative single or multiple-choice polls. All respondents were able to see the text answers of other participants once they had answered the questions. The online text-based discussion environment provided a supportive and anonymous environment for women (and people assigned female at birth) with endometriosis to share their experiences. Each individual was given a pseudonym, and the anonymity allowed the participants to unreservedly share their perspectives without fear of judgement or the risk of embarrassment.

While the discussion was open, participants logged on to the VisionsLive platform as many times as they wanted and whenever suited them. The participants were aged between 18 and 48 years and were a mixture of full and part-time workers, students, stay-at-home mothers and individuals who were not working, and the participants were a mixture of nulliparous women (who have not had children) and primiparous (who have had one child) or multiparous women (who have had one or more pregnancies). The data collected showed that the patients had experienced a broad range of symptoms and treatments.

The online environment was a requirement for conducting this qualitative and quantitative research as the period it was conducted (February to March 2022) was amid the New Zealand Omicron COVID-19 surge. In-person focus groups or discussions during this period could have been hazardous. The COVID-19 pandemic has increased the need for virtual qualitative research and platforms (22). Three key aspects of conducting online vs. in-person research, is increased data volume, reduced cost and ease of accessibility for participants (23). The online environment also aided the anonymity of the participants as their responses were not attached to their picture or name, only to a pseudonym. Additionally, the online environment allowed respondents to be drawn from a wider geographical area without the constraint of face-to-face meetings.

### Data analysis

The first step of the data analysis method undertaken was to split the quantitative and qualitative answers into two different documents. The quantitative answers from all transcripts were combined into a single spreadsheet. Similarly, the qualitative written answers from all sources were combined into a 306-page transcript. The qualitative data was analyzed through an iterative thematic approach. The transcript was coded during analysis in an inductive manner, where codes and themes were produced based on reading the patient responses, rather than being pre-conceived. The coded quotes were combined into a single spreadsheet. Quotes were re-organized into preliminary theme concepts to create an outline for the report of results. These preliminary themes were revised in an iterative manner as themes were redefined and quotes were re-organized into the final concepts of this article.

### Ethical approval

This research was approved by the University of Canterbury Human Research Ethics Committee (Ref: HREC 2022/03).

## Results

### Patient cohort

Fifty endometriosis patients participated in this research study, completing set questions, and participating in further moderated discussion. The participants were classified as having confirmed endometriosis diagnoses, or “working diagnoses” (Table 1). Having a confirmed diagnosis of endometriosis meant that endometriotic glands and stroma were histologically identified in tissue samples following surgery. Conversely, a working diagnosis meant that either the participant had not had surgery for their endometriosis, or had a negative diagnostic laparoscopy, but their symptom history meant that their general practitioner (GP) or obstetrician-gynecologist (OBGYN) concluded that diagnosis. Participants ranged in age from 18 to 48 years old, with an average age of 27.7 years. As well as including a mixture of confirmed and working diagnoses, the participants in this study cohort included a mixture of nulliparous women, primiparous and multiparous women.

**Table 1:** Patient demographics in the study cohort.

**Characteristic**	**Percentage of participants**
**Type of diagnosis**	
Confirmed by surgery	84.0%
Working diagnosis from a GP or OBGYN	16.0%
**Age**	
18–24	34.0%
25–30	40.0%
31–35	16.0%
36+	10.0%
**Parity**	
Parous	18.0%
Nulliparous	82.0%
**Work status**	
Full–time	64.0%
Studying	16.0%
Part–time	10.0%
Not working due to health issues	8.0%
Stay at home parent	2.0%
**Disease stage (*****n*** **= 42)**	
Stage I	4.8%
Stage II	19.0%
Stage III	28.6%
Stage IV	16.7%
I do not know	31.0%

**Figure 1:**
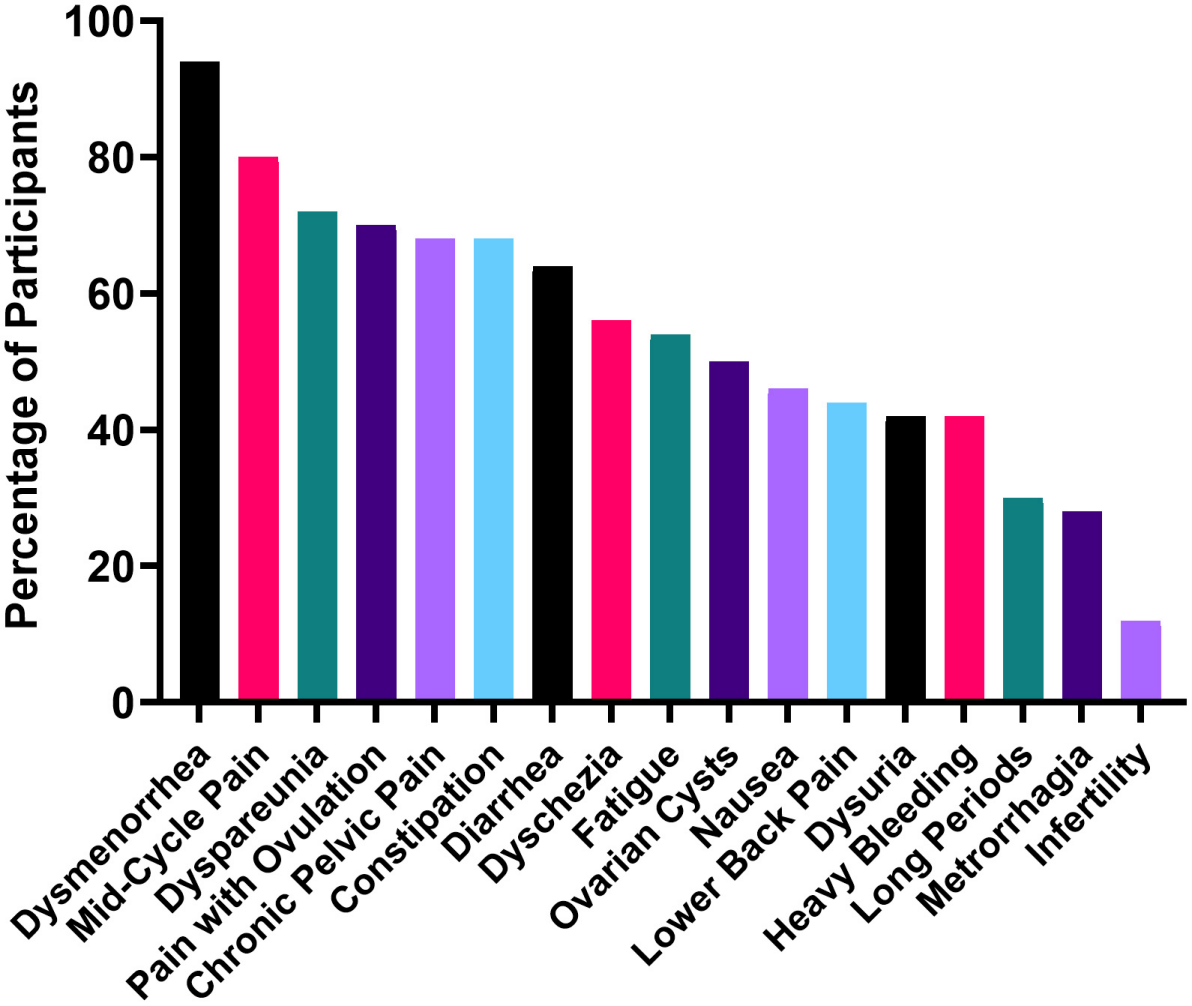
Most commonly reported endometriosis symptoms.

The participants in the study reported a variety of symptoms associated with their experience of endometriosis (Figure 1). Commonly reported symptoms included dysmenorrhea (period pain) (94%), mid-cycle pain (80%), dyspareunia (pain with sex) (72%), pain with ovulation (70%), chronic pelvic pain (for a period exceeding 6 months) (68%), constipation (68%), diarrhea (64%), fatigue (54%), nausea (46%), lower back pain (44%), and heavy (42%) and long (30%) periods. In addition to the symptoms shown in Figure 1, participants also reported brain fog (8%), insomnia (6%), depression (6%), over-sensitized skin (6%), stabbing pain up the rectum and vagina (6%), migraines (4%), “fire thighs” (4%) vomiting (4%), food sensitivities (4%), leg pain (4%), rectal bleeding (2%), joint stiffness (2%) and blacking out from pain (2%). While these symptoms were less common, they are indicative of the wide variety of debilitating experiences of endometriosis patients. In this cohort, 8% of patients were not working as a result of their symptoms.

**Figure 2:**
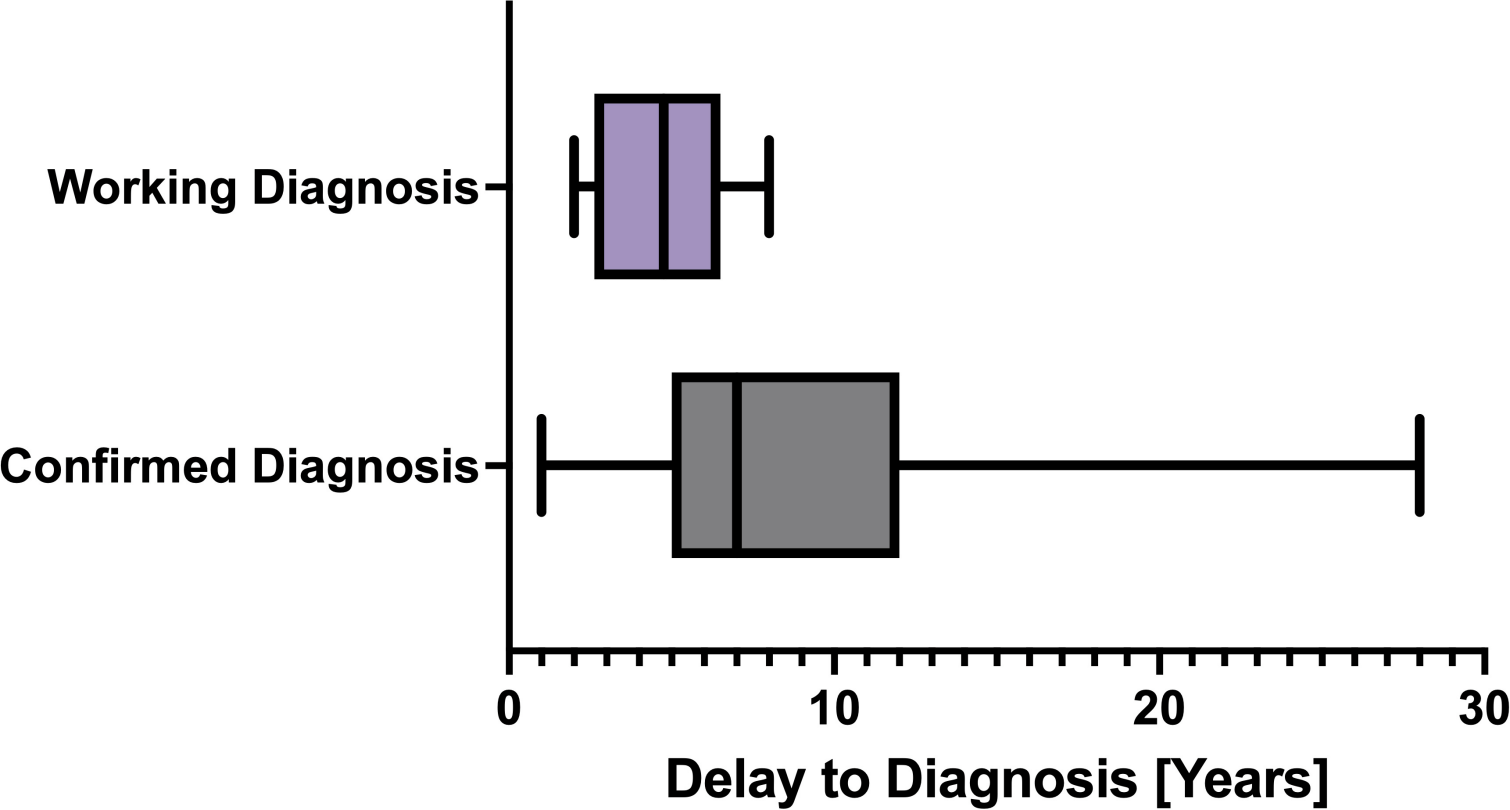
Box and whisker plot showing delay from symptom onset to diagnosis.

Among the cohort, the average age of symptom onset was 15.3 years with a standard deviation of 4.2 years and a range of 9 to 30 years of age. The average delay for the overall cohort from symptom onset to diagnosis was 7.9 years, with a standard deviation of 5.1 years, a minimum of 1 year, and a maximum of 28 years. For participants with a confirmed diagnosis, the average delay to diagnosis was 8.6 years, with a standard deviation of 5.3 years (Figure 2). The average delay was shorter, 4.6 years, for participants with working diagnoses, with a standard deviation of 2.1 years, a maximum of 8 years, and a minimum of 2 years (Figure 2). The average delay of 8.6 years for a confirmed diagnosis, is not statistically significantly different to the delay to diagnosis of 8.7 years reported in a 2022 Aotearoa New Zealand survey study which included 620 endometriosis patients (24).

### The role of pain for endometriosis patients

#### Pain intensity

Pain is subjective, and endometriosis patients do not have a method to prove that their pain is real. In an assessment of patient experiences of pain, Ahluwalia et al. identified that since painful experiences cannot be standardized, healthcare providers can perceive patients to be overreporting their pain (25). There was a range of experiences within this study's cohort of both the experience of pain and the experience of seeking relief for the pain. From the open text responses throughout the discussion about their experiences, it was evident that many of the endometriosis patients were in a pathological state of pain which manifested in a variety of ways including increased sensitivity, spasms, fatigue, nausea, as well as continuous and stabbing pains in multiple areas of the body. There was also a sense amongst patients that their pain was delegitimized due to the inability of healthcare providers to effectively observe the source of the issue. One patient (Confirmed, 25–30, Parous) recounted her experiences about what happened as soon as her endometriosis became both a palpable and observable umbilical mass: “When it got “tangible” it was easier to go to the GP about it and get things going from there.” Patients indicated not only that their experiences of pain were immense, but also that convincing others, including clinicians, of their state of pain could be challenging or impossible:

“I distinctly remember telling [my mum] that my pain was 9/10 and was only not 10/10 because I knew I was not literally dying.” (Confirmed, 18–24, Nulliparous)

“Pain was no longer just during my period, it was constant. I started struggling with pain during sex, recurring UTIs, bladder spasms, fatigue, headaches, nausea, and vomiting. Seeing as I had been dismissed by GPs in my younger years, I never mentioned it to my current GP.” (Confirmed, 31–35, Nulliparous)

“My concerns were not taken seriously until I ended up in hospital, here they found that I had an ovarian cyst rupture, when they asked why I had not come in earlier I explained that my periods were always this painful I had no idea something was actually wrong.” (Confirmed, 18–24, Nulliparous)

#### Effect in the workplace and education

Endometriosis is a condition where the prevalence, effects and costs are substantial (26). Endometriosis patients have more lost workdays than controls (27), and frequently use their sick leave for their chronic pain (28). In 2022 studies conducted in Australia and New Zealand, one in seven (29) and one in eight (24) endometriosis patients respectively, had lost their job as a result of their endometriosis condition. It was evident that for many patients within this study's cohort, the workplace was a difficult environment for them to experience endometriosis complications and symptoms. The patients within this study's cohort reported stigma, feeling unable to justify taking sick leave, worrying about the proximity of toilets, and having employers fail to understand that endometriosis is chronic, and will not suddenly go away one day:

“I was in so much pain I had to leave the meeting halfway through. I did not want to fully explain what happened to everyone, and there was a real lack of empathy.” (Working, 25–30, Nulliparous)

“When I spoke to him about getting COVID while being pregnant and hoping it will be minimal [my employer] said, ‘you'll probably get it bad, you are the sickest person I know.’ But [endometriosis] is not sickness, it is the pain and barely being able to cope, let alone work regularly.” (Working, 18–24, Parous)

“I remember one conversation with my boss when I was asking for some time off or reduced hours and he said: ‘I did not know that this would still be an issue for you’. This was after I had already spoken to him many times about it being chronic.” (Confirmed, 25–30, Nulliparous)

“I do not want other women to have to go through what I went through. It very nearly ruined my relationships, and I did end up leaving my career because of it.” (Confirmed, 36+, Parous)

Patients within this study cohort expressed the difficulties they had experienced in trying to work while experiencing symptoms, and many shared that they struggled to maintain sufficient working capacity through the pain and other symptoms. These experiences took place both in the workplace and in schooling, creating a substantial hurdle to effective performance in both environments. In a 2022 New Zealand study published in *Scientific Reports*, 23.9% of endometriosis patients had given up on their studies because of their condition, 11.0% had changed their course of study, 53.1% had delayed their exams or assignments and 66.7% reported time lost from their education as a result of their pelvic pain (24). Many patients within this study's cohort were subjected to doubt by teachers and peers during their education who believed they must be exaggerating to gain the *benefit* of skipping school or assignments:

“I struggled with support from peers in my own year group at school. I got accused by friends of making up my symptoms before diagnosis and for 'milking it' to get extensions on schoolwork post-diagnosis (excision surgery).” (Confirmed, 18–24, Nulliparous)

“I had symptoms from the moment my period first started at 11 years old, I bled through my school uniform on multiple occasions, missed a whole lot of school each month, was told I was being dramatic.” (Confirmed, 25–30, Nulliparous)

### Experiences with and perspectives of diagnostic tools

**Figure 3:**
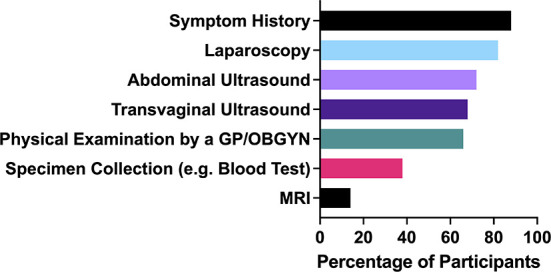
Diagnostic tests and tools utilized on the participants of the study.

There are four key diagnostic tools (Figure 3) employed for endometriosis patients: 72% of patients had been given an abdominal ultrasound, 68% had a transvaginal ultrasound, 82% had a laparoscopy and 88% had shared their symptom history with their medical practitioner. Symptom histories are where patients share with their doctors the occurrence, prevalence and degree of the endometriosis symptoms they experience. The purpose of physical exams of endometriosis patients is to assess whether bluish lesions can be identified in the vagina, to palpate the nodules in the uterosacral ligaments or pouch of Douglas, to assess if there is pain when applying tension to the uterosacral ligaments and to determine whether the uterus is retroverted (30). However, these types of physical assessments have highly variable sensitivity and specificity for the diagnosis of endometriosis, even when carried out in expert centers (30).

There are no biomarkers in blood with sufficient efficacy to diagnose all forms of endometriosis (31). The only endometriosis-related blood test highlighted by a patient in this cohort was a test for elevated CA-125. Tests for CA-125 (cancer antigen 125) have been assessed as a diagnostic metric for endometriosis (32) but this test has a very low sensitivity of only 28% with a specificity of 90% in blood serum (33). Testing for combinations of biomarkers in blood serum, urine (33), peritoneal fluid (34) and follicular fluid (35) may be promising in the future but were not reported in the experiences of patients within this cohort.

#### Ultrasonography

Ultrasound scanning works by emitting sound waves and then recording the waves that echo back. Abdominal and transvaginal sonography is the first step for investigating suspected endometriosis in the New Zealand Diagnosis and Management guidelines for endometriosis (13). Stage I (small patches and surface lesions) and stage II (more and deeper implants) endometriosis are not visible during ultrasound examinations, stage III (more widespread disease starting to infiltrate tissue, often scarring and adhesions) is sometimes identifiable, and stage IV (affects most pelvic organs, often with adhesions and anatomical distortion) is usually visible during this imaging (13).

There are two key issues with ultrasound imaging for endometriosis diagnosis. The first issue is that ultrasound scanning is highly operator-dependent, and scanning for endometriosis often requires particular techniques to be able to effectively locate and identify abnormalities (36). This was highlighted in one participant's (Working, 18–24, Nulliparous) experience who said: “Abdominal and transvaginal ultrasound was pretty horrible. I had a trainee doctor, and nothing against trainee doctors, I felt like I could not say no. I wish [I had asked for] a fully trained radiologist, I think the outcome may have been different.” Secondly, of the three sub-types of endometriosis, only ovarian endometriosis and deep infiltrating endometriosis are likely to be identified during an ultrasound scan, whereas superficial endometriosis often eludes detection *via* ultrasonic imaging (36). The experiences of participants within this cohort varied concerning abdominal ultrasound. Some had very positive experiences, while others had negative experiences, usually when they felt dismissed, or when nothing could be identified on the scan:

“Abdominal ultrasound: was probably the best diagnostic tool that I've experienced. While in hospital I was able to see what was happening inside of me. And then 7 weeks later it also provided a clear image.” (Confirmed, 25–30, Nulliparous)

“I was also booked in for an ultrasound at [a radiology clinic], this was easily one of the worst experiences of my life. The nurse there was incredibly dismissive of my fears and anxieties surrounding the procedure and made me feel scared and embarrassed.” (Confirmed, 18–24, Nulliparous)

Transvaginal ultrasound can be an effective diagnostic tool for ovarian endometriosis, with a sensitivity of 93% and 96% specificity (36), and deep infiltrating endometriosis, with a sensitivity of 76% and 94% specificity (37). Unlike abdominal ultrasound, transvaginal ultrasound experiences were consistently negative throughout the cohort, who found the experience painful at best and traumatizing at worst. Transvaginal ultrasonography involves the insertion of a probe 5–8 cm into the vagina (38). According to the literature, transvaginal ultrasonography is not expected to be painful, but may cause some discomfort upon insertion, but should not cause any discomfort following the conclusion of the scan (39), and in most cases is an even more comfortable experience for patients than abdominal ultrasonography (40). Conversely to the literature, multiple participants in this study cohort shared that the experience with transvaginal ultrasound caused them to cry during the procedure, and some explained they were in pain days afterwards:

“Everything was within the realm of normal they said. But I was crying on the table with the transvaginal ultrasound and was still uncomfortable and in pain days later. Nudging my organs felt like my insides were being ripped. I do not know how much of this was down to inexperience [of the ultrasound operator], or my anatomy. Either way, I got next to nothing out of that experience.” (Working, 18–24, Nulliparous)

“I was forcefully held down during on at [the hospital] even though I was screaming at them to stop [the transvaginal ultrasound].” (Confirmed, 18–24, Nulliparous)

“The ultrasounds were horrible, the hospital initially gave me a guy to perform them which made me really stressed, but I asked if I could please have a female, especially for the transvaginal ultrasound and the hospital agreed except then I had to wait for quite a while and then I don't think she was very gentle, it was really painful.” (Confirmed, 25–30, Nulliparous)

#### Laparoscopy

Eighty-two percent of the participants in this study had surgery to diagnose their endometriosis. Laparoscopic surgery is considered the “gold standard” for the diagnosis and treatment of endometriosis (5) and is the only method available to “confirm” endometriosis histologically (41). Endometriotic lesions have a vast range of appearances and can look black, brown, blue, clear (42), red, white, fibrotic, fatty or smooth (43). This variation in appearance can lead to lesions being confused with non-endometriotic growths, thereby allowing endometriosis to be missed (43). In this cohort, endometriosis patients were primarily relieved to have a definitive diagnosis following laparoscopy, but found recovery from the surgery challenging, and wished there was more post-operative support. For some patients, during their first (or only) laparoscopy, no endometriosis was found. In one case, it was evident that the original surgeon lacked the expertise to locate and remove the endometriotic lesions. This was evident since not only was endometriosis expertly removed during the second laparoscopy, but her surgeon also highlighted the presence of endometriosis on the images from the patient's first surgery. The range of experiences with laparoscopy were articulated by the patients within this study:

“Laparoscopy was life-changing in a way that it provided a definite diagnosis, and I began being taken seriously with my symptoms. I did notice relief from post-surgery.” (Confirmed, 18–24, Nulliparous)

“My recovery after the second surgery was pretty brutal. I had a lot of nerve damage. I was recovering from it as well as dealing with the Mirena side effects. Once those calmed down, my pain was lessened, and I started to have a relatively normal life again.” (Confirmed, 25–30, Nulliparous)

“When my surgery found nothing, and it fixed nothing, I felt so angry and confused, because I had no answers, and they didn't remove any [endometriosis], so I knew nothing would get better. It was frustrating, and all I could do was cry, because the pain would not be going away.” (Working, 18–24, Nulliparous)

#### Symptom history

Working diagnoses are primarily given to endometriosis patients by a GP or OBGYN based on their history of symptoms. The definition of a working diagnosis of endometriosis is that a patient is suspected by their GP or OBGYN to have endometriosis because of their symptom history, but surgical intervention has not been attempted for a diagnosis, or, as in the cases above, endometriosis has been missed during surgery. For many patients, being assigned a working diagnosis was initially a cathartic experience as it was evidence that a doctor was willing to believe them. As one patient (Working, 25–30, Nulliparous) in this study elaborated: “It is such a long process and while you are waiting for all of these appointments and doctors to believe you, you are still experiencing intense pain! To get some sort of answer is definitely a relief, but it totally sucks that they have to actually cut into you to make it conclusive.”

While a working diagnosis usually means the patient is working with their primary healthcare practitioner to pursue treatment. Some participants with working diagnoses expressed that they feel excluded from endometriosis forums where other patients have surgically confirmed diagnoses, as they feel like less legitimate patients. Sixteen percent of patients within the study cohort had working diagnoses of endometriosis, but the proportion of patients in New Zealand that will have working diagnoses will likely be larger in the future. This is because the Endometriosis Management and Diagnosis guidelines in New Zealand dissuade medical professionals from pursuing diagnostic laparoscopy for endometriosis patients for the primary purpose of confirming the diagnosis (13). This is part of an international trend as the European Society of Human Reproduction and Embryology 2022 guidelines for Endometriosis similarly dissuades surgical diagnosis of endometriosis moving the focus to pain management and hormonal medications (44). Both New Zealand and European guidelines only suggest surgical treatment of endometriosis when non-surgical treatments have been unsuccessful or are inappropriate. While working diagnoses are likely to become increasingly common, endometriosis patients within this cohort indicated that they were still prone to dismissal by both health practitioners and themselves because of the lack of histological confirmation:

“As someone with a ‘working diagnosis’, I have been really frustrated with the health care responses I've experienced. I know others have similar experiences.” (Working, 25–30, Nulliparous)

“There are still doubts in my head that make me think I am just making it all up.” (Working, 18–24, Nulliparous)

“I have like imposter syndrome, having a 'working diagnosis', but not an actual diagnosis, I do not really feel like I can participate in the [endometriosis] community.” (Working, 18–24, Nulliparous)

Prior studies into the impact of endometriosis diagnoses on patients, found that confirmed diagnoses gave patients a language to discuss their disease, offered management strategies and confirmed that their symptoms were not the result of cancer (45). While the emphasis on working diagnoses may allow more patients to feel their pain is more legitimized, the lack of *total confirmation* prevents patients from feeling able to fully adopt the label of “endometriosis patient.” One patient (Confirmed, 18–24, Nulliparous) explained: “I think a diagnosis was the most helpful thing, from the very beginning just knowing what I was experiencing was not normal and it was not my fault and [I was] being heard.”

#### Emotions patients exhibited in response to their endometriosis diagnosis

**Figure 4:**
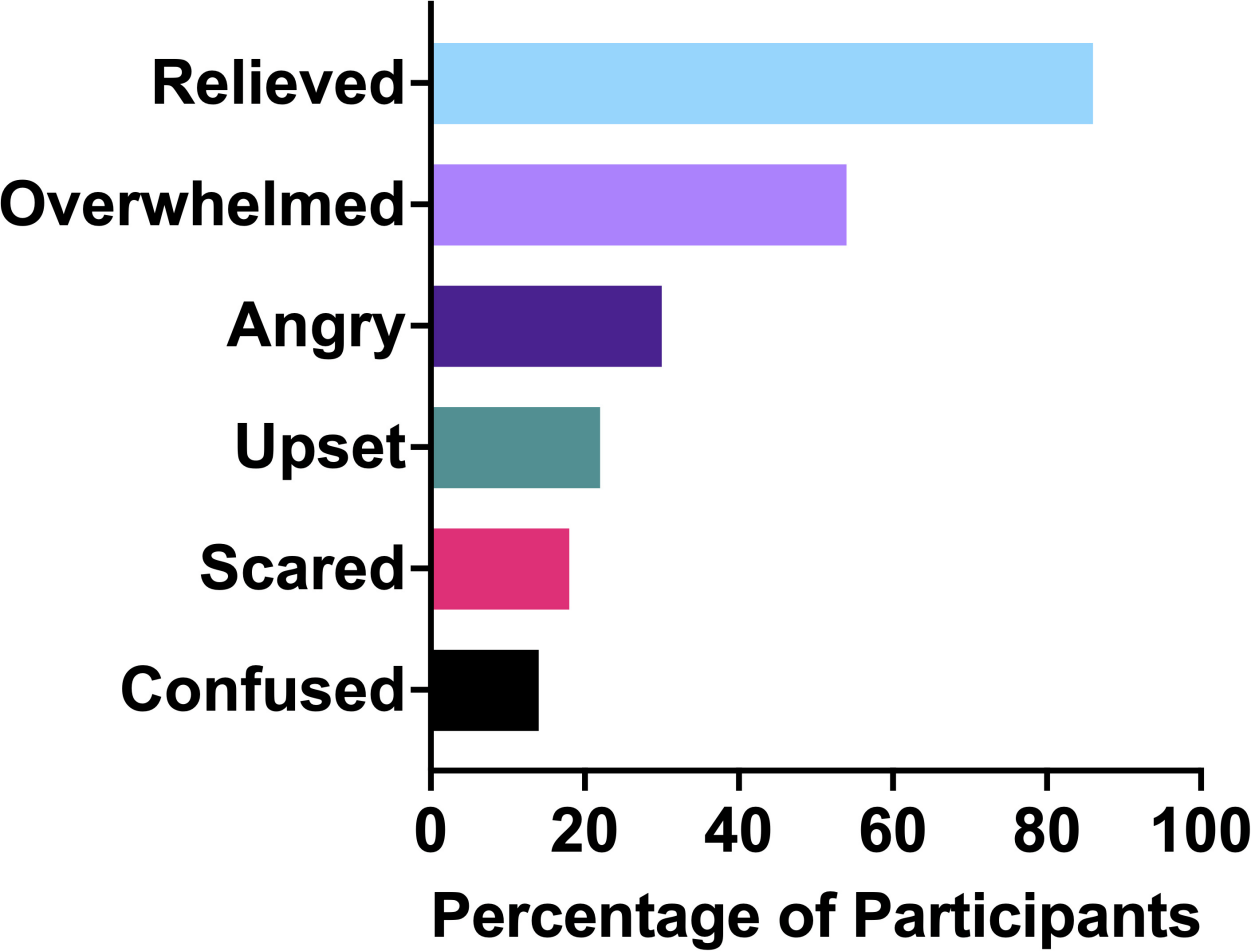
Emotions endometriosis patients felt upon receiving their diagnosis.

Endometriosis diagnoses can elicit a vast range of reactions and emotions. In this cohort, the most common emotion (Figure 4) was relief (86%). The next most common emotions were overwhelmed (54%) and angry (32%). Other emotions participants shared they had experienced were disappointment, happiness, shock, validation, tiredness, sadness, vindication and affirmation. Relief is objectively a strange reaction to finding out one has a chronic, invasive, inflammatory disease with no cure. Endometriosis patients are often made to feel immense doubt about their own experiences, which means that the confirmation that something palpable was occurring in their body, and not just in their head, caused considerable relief:

“The surgeon told me I had aggressive stage 3 [endometriosis] and I cried with happiness. Everyone was so confused. But I had been in severe pain for 7 years and I finally knew it was not all in my head.” (Confirmed, 25–30, Nulliparous)

Relief was also often accompanied by other emotions such as concern, sadness and anger. In addition to relief, many patients shared that they felt vindicated or validated, that they had been right about having endometriosis, and those that had dismissed them had been wrong. Endometriosis is a fundamentally incurable disease (46). It is resistant to many treatment options. It often recurs following surgery (47). It can turn patients into chronic pain sufferers, while also lowering their pain tolerance (4). Many cases will never be identifiable on scans (13) and in some cases, endometriosis can even be missed during surgery (43). It is a debilitating condition that primarily affects young women in the prime of their life. Thus, it is unsurprising that 54% of the participants in the study felt overwhelmed by their diagnosis. Anger was another key emotion amongst the cohort, with 32% reporting anger as an emotion they felt in response to their diagnosis. The patients were angry primarily at the doctors who had dismissed them or prolonged their journey to obtaining a diagnosis. Anger was also targeted toward the pain they had gone through, the lack of a cure for endometriosis and for some, the fact that their experience could have been even worse. Anger was often part of a mixture of emotions patients felt upon diagnosis, along with validation and feeling overwhelmed:

“I was relieved that the time I had spent making sense of my condition and grieving my health was validated, overwhelmed that I had put up with so much over the years and accepted that to be normal, and angry about the social conditions that have allowed this to happen and especially to other people who may not be in as fortunate as a position as I was to access healthcare.” (Confirmed, 18–24, Nulliparous)

“Relieved that finally I had an answer after being tossed aside and gaslighted by many doctors, teachers, co-workers, the list goes on. Anger for the above. Anger for living through so much pain when I had told someone at 16–if I had been properly treated then, maybe I would not have lived through so much pain.” (Confirmed, 18–24, Nulliparous)

Twenty-two percent of patients within the study cohort stated that their diagnosis made them feel upset, with others also stating they felt disappointed or tired. These endometriosis patients were upset over misconceptions about endometriosis, their delays to diagnosis, and the lack of perceived remaining options for them. Those that talked about how their endometriosis diagnosis had made them feel scared explained that those feelings were often derived from the information they were given about the associations between endometriosis and infertility. Feeling upset could also result from a perceived lack of remaining options for care, when existing options had failed:

“Now I am mainly tired, and I am losing hope that things will get better. When I first got the diagnosis there were treatment options we could pursue. We have pretty much tried everything now.” (Working, 18–24, Nulliparous)

Endometriosis is a chronic pain condition that can have a substantial negative effect on the mental health of patients due to both symptoms and impacts on social relationships and sexuality (48). In a 2021 study of 79 Croatian endometriosis patients, 44.3% presented with depressive symptoms, 31.7% with stress symptoms and 25.3% with symptoms of anxiety (49). Endometriosis patients within this study's cohort highlighted the toll endometriosis could take on their mental health through anxiety over the return of symptoms, lack of intimacy with partners because of pain, feeling judged by others and the physical toll of hormonal medications on their bodies. However, the danger of medicalization of these mental health struggles was highlighted by one patient (Confirmed, 36+, Parous) who was misdiagnosed with premenstrual dysphoric disorder instead of endometriosis: “The inclusion of [Premenstrual dysphoric disorder] on the [Diagnostic and Statistical Manual of Mental Disorder, Fifth Edition (2013)] is also incredibly problematic as it basically labels women with painful periods and other symptoms as having a mental health condition.” Endometriosis has impacts on all aspects of quality of life and wellbeing. In a 2022 New Zealand-based study, 77.3% of patients indicated their chronic pelvic pain had negatively impacted their social relationships (24). In this cohort, patients indicated their mental health was negatively impacted by the fear of recurrence and the disruptions to their social and sexual relationships:

[I am] “doing okay post all that [two surgeries] but [I] have a lot of anxiety around when it could rear its ugly head again and disrupt my whole life again.” (Confirmed, 18–24, Nulliparous)

“Sexual dysfunction is another ongoing issue (I have not had intercourse or intimacy with my long-suffering and supportive partner for over a year) and I am deeply depressed, lost and confused because I have no control over my body, the pain, the symptoms that seem to live on.” (Confirmed, 18–24, Nulliparous)

### Patient experiences with treatments

The frontline treatment for endometriosis is hormonal medications, primarily in the form of a combined oral contraceptive pill (COCPs) with estrogen and progesterone, progesterone-only pills (POPs), intrauterine devices (IUDs) and hormone replacement therapy (HRT) (17). To cope with the symptoms of pain associated with endometriosis, patients are frequently prescribed pain killers, primarily NSAIDs (41), which have known health issues with long-term use. When front-line methods to repress endometriosis symptoms are unsuccessful, laparoscopy and other surgical interventions are employed.

#### Hormonal medications

The purpose of using hormonal medications for endometriosis treatment is to repress endometriotic lesions and/or to prevent the menstrual cycle (16). Many women with endometriosis cannot use hormonal medications because the side effects [such as nausea, weight gain and capacity to worsen depression (17)] are too severe or because of a desire to get pregnant. In this cohort, as shown in Table 2, 80% of participants had been prescribed and used COCPs, 64% had used an IUD and 50% had used POPs. While hormonal medications are the first line prescription for endometriosis, only 25% of participants who had used COCPs found them effective, and 24% using POPs found their use effective. One participant (Confirmed, 18–24, Nulliparous) who shared a particularly positive experience of POPs shared: “Progesterone only pill has been a game-changer for me. They do not always work well, and I have been told that they basically work until they do not and then it is on to the next one, however for the most part they play a huge role in managing my symptoms as they stop my body from cycling altogether which I think is the best management suited to me given my lifestyle and career.”

**Table 2:** Distribution of participants that used a treatment and considered that treatment effective.

	**% Of participants that have used the treatment**	**Number of users**	**% Of users that found the treatment effective**
Pain relief medication	96%	48	46%
Laparoscopic surgery	84%	42	67%
Combined oral contraceptive pill	80%	40	25%
Exercise	66%	33	45%
Intrauterine device	64%	32	50%
Progesterone–only pill	50%	25	24%
Neuropathic pain relief	44%	22	50%
Counseling	40%	20	20%
Altered diet	16%	8	50%
Hysterectomy	12%	6	100%
Hormone replacement therapy	12%	6	33%
Fertility treatments	10%	5	20%

**Figure 5:**
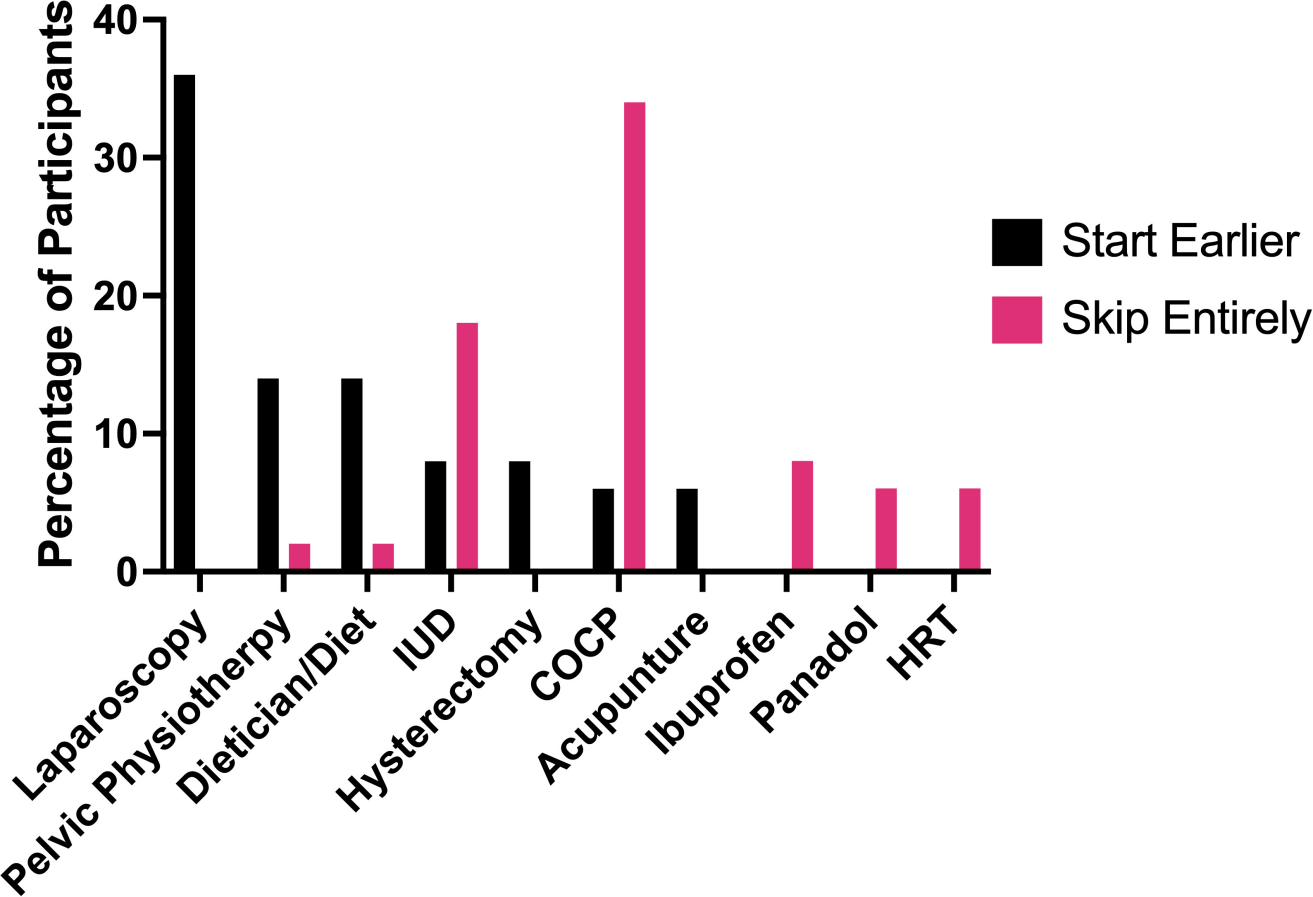
Treatments participants stated they wished they had started earlier or skipped entirely.

When asked which treatments they would have liked to have started earlier, only 6% said they would want to go onto the pill earlier, while 34% said they would want to skip using the pill entirely (Figure 5). Prescription of COCPs and POPs was so prevalent amongst the cohort of this study that even as pre-teens and for patients experiencing extreme symptoms these hormonal medications were given as the first-line solution. In New Zealand, the 2020 guidelines for diagnosis and management of endometriosis suggest that hormonal medications should be the “first line” treatment, primarily progesterone-only therapies unless the patient is trying to become pregnant (13). Some of the patients shared that not only were hormonal medications the main or only treatment option they were offered, but it may have caused further pain or acted as a mask for their endometriosis, preventing symptoms for a time, but not eliminating the issue long-term:

“It took 9 years to get a diagnosis in that time I was told it is just a bad period, contraception will stop everything.” (Confirmed, 31–35, Parous)

“During the wait, I went on the [Depo Provera] injection which unfortunately worsened my pain (which was later diagnosed as vulvodynia) this was discovered when we attempted a cervical smear test in a clinic where I was screaming in pain and then passed out.” (Confirmed, 18–24, Nulliparous)

“The pill only masked the issue and allowed doctors to keep pushing it on me and not take me seriously. Trying to ‘push through it’ only caused me more pain.” (Confirmed, 18–24, Nulliparous)

“I tried a lot of contraceptives, which was always treated in a very banal way by medical professionals, but I struggled quite a bit with being on birth control. The pill had horrible mental side effects, and all other birth control methods caused my periods to become incredibly long and close together… The impacts of birth control on my body were quite stressful and physically exhausting as well, but it was never treated as something out of the ordinary… birth control never felt like treatment to me, it only felt like treading water, and it never worked sustainably.” (Confirmed, 18–24, Nulliparous)

IUDs are contraceptive devices inserted into the uterus through the cervix which deliver Levonorgestrel (a synthetic progesterone). There are two hormonal IUDs available in New Zealand, the Mirena and the Jaydess. The Mirena releases an average of 15 μg of hormone every 24 h (13) and lasts an average of 5 years (50), while the Jaydess releases 13.5 μg every 24 h (13), lasts up to 3 years and has a smaller insertion tube diameter (50). The IUD is a suggested treatment for endometriosis for the consistent delivery of synthetic progesterone and is frequently placed during surgery as a method of post-operative care. However, consistently within this cohort, the patients shared their negative experiences with their IUDs, and many found that having their IUDs removed brought them relief from pain:

“The Mirena was awful. It caused horrible cramping that felt like I was being stabbed repeatedly. I was very frustrated that I had told my gynecologist that my mother had (painfully) ejected TWO IUDs, yet she still assured me that they would not be a problem for me. It turned out that the IUD was placed correctly, and my uterus just hated it.” (Confirmed, 36+, Nulliparous)

[I had a negative experience with my] “IUD. [It] caused worse pain than I was experiencing with [endometriosis] beforehand.” (Confirmed, 31–35, Nulliparous)

Within this cohort, 64% of the patients had tried treating their endometriosis symptoms with an IUD, but only 50% of these patients found it an effective treatment. When asked which treatment they would wish to start straight away, 8% of participants shared they would ask for an IUD, while a further 18% stated they wished they had never used one. One positive statement about the IUD stated (Confirmed, 18–24, Nulliparous) “[the IUD] kept the symptoms at bay for the most part of four years”. Some participants highlighted that they had painful experiences during their insertions. New Zealand guidelines suggest health practitioners recommend that their patients take acetaminophen (aka Paracetamol, Panadol, or Tylenol) or ibuprofen (aka Advil or Motrin) before the procedure, and take NSAIDs after the insertion if they experience pain (51). Some participants strongly rejected this advice and found it insufficient to counter the pain of their experience:

“Also, IUD insertion needs some sort of anesthetic. Panadol just does not cut it. The doctor lies and tells you it's relatively painless and that you will have 'mild discomfort'. The nurse warns you that you need to take more than a couple of Panadol.” (Confirmed, 36+, Nulliparous)

#### Pain relief medication

Fundamentally, endometriosis is a condition of pain, however, there is inconclusive evidence that NSAIDs, such as aspirin and ibuprofen, provide greater relief to endometriosis symptoms than placebos (52). Opioids are not a recommended treatment for endometriosis (53), but 89% of endometriosis patients in a US cohort were utilizing them for managing their pain (27). Within our study's cohort, some participants highlighted how ineffectual they found pain relief options to be, the lengths they had to go to access pain relief that would allow them to go about day-to-day activities, and the insistence some faced from doctors who assured them options such as acetaminophen would be sufficient. While 96% of patients had tried pain relief for their endometriosis, less than half (46%) found that medication effective. Furthermore, multiple patients indicated they wished they had skipped using ibuprofen (8%), Panadol (6%) and “strong pain medication” (4%) entirely. The low reports of effectiveness align with the written reports of this cohort's patients who reported that pain relief medications were insufficient for their pain, negatively impacted their mental health, or caused immense unintended side effects:

“I went back to my GP and he prescribed some Panadol. I then went to a different GP, who told me that since I had already been given the pill and Panadol she said there was nothing else to do.” (Confirmed, 25–30, Nulliparous)

“I found that Panadol and Ibuprofen were not that effective, and I got sick of being told to take pain relief.” (Confirmed, 18–24, Nulliparous)

“Being on opioid pain relief for roughly a year took a pretty big toll on my mental health. I hated feeling dependent to have a sort of normal life.” (Confirmed, 25–30, Nulliparous)

“Hot water bottles helped but I ended up burning my back (2nd-degree burns) because the codeine made me sleep soundly and I did not wake up when the [hot water] bottle leaked.” (Surgical, 36+, Nulliparous)

##### Gastrointestinal risks

Long-term use of NSAIDs has known effects on the digestive tract including esophagitis and esophageal strictures, acute erosive gastritis, bleeding, gastric ulcer, upper gastrointestinal bleeding (54), small intestine ulcers, non-specific colitis, and exacerbated symptoms for ulcerative colitis and Crohn's disease (55). In the United States, gastrointestinal tract complications from using NSAIDs are the most commonly reported drug side effect and increased time of use increases the risk of an adverse event (56). There has been evidence to suggest that 14–25% of NSAID users may develop gastric and duodenal ulcers due to their use (55). Patients within the cohort highlighted concerns about depending on pain medication to function in their day-to-day lives, being viewed as drug seekers, facing physical ramifications due to prolonged NSAID use or fearing those ramifications:

“My first symptoms were when I got my first period at age 13. I had excruciating cramps, and my mother gave me Ibuprofen. It helped, and I continued to take Ibuprofen for about 17 years until my stomach could not handle it anymore.” (Confirmed, 36+, Nulliparous)

##### Medicinal cannabis

In a 2021 retrospective cohort study of 252 endometriosis patients using cannabis, 57.3% were using it to treat their endometriosis-related pain (57). Patients reported that cannabis use was effective for their pelvic pain, gastrointestinal issues and mood (57). In a study of Australia and New Zealand-based endometriosis patients using cannabis for their pain it was reported that 72.0% and 88.2% of patients respectively, were illicitly obtaining cannabis to treat their endometriosis symptoms (58). In the same study, only 23.1 and 5.9% respectively were able to obtain medicinal cannabis through a doctor's prescription. Among the cohort of the published study, cannabis use resulted in a substantial reduction in patient use of opioid and non-opioid analgesics, hormonal therapies, neuropathic pain relief, antidepressants, and antianxiety medications (58).

In a study of New Zealand endometriosis patients using cannabis to treat their endometriosis-related symptoms, 81.0% had reduced pain, 79.0% had improved sleep and 81.4% reported their cannabis use had allowed them to reduce their use of other medications (59). The reduction in the use of other medications when using cannabis as a self-management strategy is a consistent trend in recent literature. In a 2020 Australian study, 56% of endometriosis patients using cannabis for symptom management were able to reduce the use of pharmaceutical medications by at least half (60). According to the Cannabis Clinic, THC will cost a patient $1.00 to $2.00 each day, but there is a limited selection of products available for purchase (61). The cost of CBD oil varies from $5.80 to $7.60 each day (61). These prices would mean that a year of THC use would cost $365 to $730, while CBD oil could cost $2,117 to $2,774. Within this study cohort, every patient who mentioned the use of medicinal cannabis to treat their symptoms spoke positively of the experience, and one highlighted it as the treatment they wished they had started straight away:

“Medicinal cannabis (CBD and THC oil) has really helped with the pain and allowed me to get on with my day most of the time… The best care I received was at the cannabis clinic where they were full of empathy and more helpful on my pain related to [endometriosis] than my own GP.” (Working, 25–30, Nulliparous)

There is some evidence to suggest that the use of cannabis can be associated with stroke and atrial fibrillation, can alter the brain by reducing glutamate, decreasing the hippocampal volume and causing poorer global functioning, (62) and when consumed by pregnant users can increase the risk of low-birth-weight and other neonatal complications (63). In a 2021 Danish study of 2,841 users of cannabis for medicinal purposes, 85.5% reported that they considered the side effects of prescription drug use to be worse than those of cannabis (64). While the positive experiences of patients is positive, it was recognized in the Danish study that the long-term consequences on both physical and mental health of using cannabis for medicinal purposes requires further research (64).

##### Neuropathic pain relief

Amitriptyline (aka Elavil or Vanatrip) is a tricyclic antidepressant, which can be used to treat neuropathic pain and prevent migraines (65). Treating pelvic pain with amitriptyline uses a much lower dose than the average antidepressant dose, 5–25 mg daily compared to 150 mg (66). Neuropathic pain relief, such as amitriptyline, is considered a pharmacological treatment option for the central sensitization of pain that can stem from endometriosis, as well as treat any other chronic overlapping pain conditions, such as vulvodynia (chronic pain at the opening of the vagina), that the patient may have (67). Forty-four percent of the patients within this study's cohort had used neuropathic pain relief as a treatment, with 50% finding it to be an effective treatment. This was one of the third highest-rated treatment for effectiveness for users behind hysterectomy (100%) and laparoscopy (67%). Neuropathic pain relief exceeds the percentage of users who found pain relief medications (46%), HRT (33%), COCPs (25%) and POPs (24%) effective. In this study cohort, 4% of patients wished they had started taking amitriptyline when they started having endometriosis symptoms. As with other treatment options, amitriptyline also has side effects including feeling sleepy or faint, having a dry or sore mouth, constipation and/or worsening depression (65); however, none of these symptoms were mentioned as being of concern by the patients during the study. The perceived effectiveness of neuropathic pain relief was indicated by patient reports in the study such as:

[Because of amitriptyline, having endometriosis] “does not haunt the majority of my days now.” (Working, 18–24, Nulliparous)

#### Laparoscopy

The purpose of laparoscopic surgical treatment for endometriosis is to excise endometriotic lesions. These lesions can then be histologically assessed to check the excised tissues for characteristics (such as endometrial glands and stroma in the excised tissue) that confirm the presence of endometriosis. While surgery is the only currently available treatment to remove endometriotic lesions, and hopefully by extension endometriosis symptoms, it is often not a permanent solution. In one study, 42% of the 1,160 endometriosis patients included had had at least three surgeries to treat their endometriosis (68). Endometriosis is known to be able to recur following surgical treatment, both in terms of symptoms, and the endometriotic lesions themselves (7). This recurrence occurs in 40–50% of patients (47) and can exacerbate both pain and fertility issues (69).

Despite issues of recurrence, the endometriosis patients within this cohort had primarily positive experiences with surgical treatment for their endometriosis, even if the relief was temporary. Some participants raised a key concern that they were not provided with sufficient support following surgery to heal successfully, particularly multiple participants who found their cramping worse than ever in the months following surgery, before beginning to feel relief. Of the 84% of participants that had had laparoscopic surgery for endometriosis, 67% found it to be an effective treatment. Laparoscopy was the highest-rated treatment that the patients within the study wished they had had straight away (36%), and none stated they wished they had skipped it. Even when the experience of laparoscopy was challenging or painful, many patients still considered it an overall positive experience:

“The laparoscopy was what finally gave me a few years of peace. I was lucky enough to have a surgeon that actually knew what he was doing and could also tell before the surgery through ultrasound/touch on my sensitive areas where the worse affected area was.” (Confirmed, 18–24, Nulliparous)

“Although the surgeries and recovery were nerve-wracking and I had hesitations about them, it was a positive experience in terms of the outcome.” (Confirmed, 31–35, Parous)

“Surgery did not help with my pain at all. Although knowing I had endometriosis and improved fertility from the surgery was enough for me.” (Confirmed, 25–30, Nulliparous)

#### Hysterectomy

Endometriotic tissue is similar to the tissue that lines the uterus. The partial or complete surgical removal of the uterus during a procedure called a hysterectomy may remove a source or area of endometriotic tissue that causes the condition. Having a hysterectomy means the patient no longer has menstrual periods, but the procedure alone does not cause menopause. Surgical menopause is induced by bilateral oophorectomy, where the ovaries, which are the main source of estrogen for the body, are removed. Hysterectomy for endometriosis must be performed simultaneously with the excision of lesions, or the risk of persistent symptoms is increased (70). Hysterectomy has also been shown to be an effective treatment for endometriosis, through simultaneous removal of extra-uterine endometriotic lesions and the removal of adenomyotic lesions from the uterine muscle. Adenomyosis is a condition separate from endometriosis where endometrial tissue grows into the muscle of the uterus, and can only be cured by hysterectomy (71). Curing adenomyosis does not require an oophorectomy (71). While not a definite cure for endometriosis, a Swedish study of 5,482 endometriosis patients who underwent a hysterectomy found that 91.3% of the women were satisfied or very satisfied with their hysterectomy, while 95.2% said their medical condition was improved or much improved (72).

In this cohort, 12% of the endometriosis patients had undergone a hysterectomy, and 100% of these patients had found it an effective treatment for their endometriosis. Some of the patients shared that they had had to convince their medical practitioners to support their ambition to obtain a hysterectomy. Concerns of medical practitioners about the medical procedure ranged from it will make you die young” to “how will your family line continue?” and “what if you meet someone and they want kids?”. Some patients expressed massive relief following the procedure, with some wishing they had had the procedure at a younger age and avoided pain:

“Endometriosis was not even considered in my case until after I had everything removed and my surgeon told me that that is what he found. Surely an exploratory operation before a full hysterectomy could be worth it for some women?” (Confirmed, 36+, Parous)

“One day many months after the [hysterectomy], I woke up with no pain and thought something was wrong. And cried. This is what no pain feels like. New me. Not angry at the world anymore… This is a massive decision to make, means no babies, but my mental health welcomed it.” (Confirmed, 36+, Nulliparous)

“The relief, the liberation, it has been life changing. Getting rid of my reproductive organs has been hands down the best most liberating decision of my life.” (Confirmed, 25–30, Nulliparous)

#### Alternative treatments

The impact of endometriosis on the lives of patients are complex and are not simply an interruption of psychosocial interactions and relationships (73). Endometriosis patients in a qualitative Australian cohort displayed complex health-seeking patterns which included moving in and out of medical care, particularly before an official diagnosis was made (74). Endometriosis patients tend to become experts about their condition in order to determine methods of self-management (75) or to apply a patient-centered approach with complementary treatments (76). Within this study, cohort patients reported their experiences with alternative therapies such as exercise, physiotherapy, botulinum toxin, diet changes, and acupuncture.

##### Exercise and physiotherapy

Exercise and increased physical activity are often suggested as an alternative to treatment methods such as hormones, pain relief and surgery (77). The principle behind regular physical activity having protective effects for endometriosis is that this exercise can induce an increase in systemic levels of anti-inflammatory and antioxidants (78). However, a 2021 systematic review found that there was inconclusive evidence to justify this treatment approach (77), as did an earlier systematic review in 2014 (78). In this cohort, 66% had tried exercise with 45% of these patients finding it an effective method for treating their endometriosis symptoms. Of those who found it effective, 4% said it was a treatment they wished they had started right away, while others also suggested stretching and yoga as an approach to managing their pain. Other participants had more negative experiences and found that exercise tended to exacerbate their endometriosis pain:

“I am a fit person. Yet [exercise] resulted in extreme pain frequently and was not helpful.” (Confirmed, 18–24, Nulliparous)

Pelvic physiotherapy was well-regarded by this study's cohort, with only one participant wishing they had never tried it to treat their endometriosis, while 7 wished they had started right when they started having endometriosis symptoms. Of the 18% of the cohort who had tried pelvic physiotherapy to treat their endometriosis symptoms, 89% had found it to be effective for them. The purpose of pelvic floor physiotherapy is to retrain the pelvic muscles so that they can relax and coordinate contractions. The expectation is that this improved capacity to relax and co-ordinate pelvic floor muscles will improve patient experiences of pelvic and back pain, painful urination, and bowel movements (79), as was indicated by some patients in this study:

“Pelvic floor [physiotherapy], amazing, helped me in so many ways for pain management, relationships/sex.” (Confirmed, 25–30, Nulliparous)

##### Botulinum toxin

Botulinum toxin (BTX) injections are an emerging potential treatment for endometriosis symptoms (80, 81). Pelvic floor muscle spasms have been identified as a key pain focus for endometriosis patients and may be a mechanism for initiating or sustaining sensitization (82). A 2021 pilot study that injected BTX *via* hysteroscopy into the uterine myometrium (muscle) in patients with severe dysmenorrhea and pelvic pain, found that quality of life scores “improved dramatically” and sexual activity discomfort reduced significantly (80). After 6 months, 40% of the 30 patients returned for new injections, while a further 47% had not had a reappearance of symptoms at 6 months and thus did not need a further injection (80). Although longer-term cohort studies are still required, the initial results for this treatment approach are promising. Another study found that within 4–8 weeks after a post-transvaginal BTX injection into pelvic floor muscles, there was reduced pain for all participants and reduced or undetectable spasms in all patients that had had palpable spasms prior to injection (81). Only a few endometriosis patients within this study cohort had trialed BTX injections for their endometriosis pain, however, their experiences with the procedure were positive, and other participants considering the treatment were excited at the prospect:

“Pelvic Botox [and] nerve blocks have been incredible for me, Botox reduced [the] pain.” (Confirmed, 25–30, Nulliparous)

##### Dietary changes

Imbalances to gut microbiota composition have been connected to the compromised immunosurveillance and altered immune profiles associated with endometriosis (83), with rodent studies consistently showing the impact of the gut microbiota on endometriosis and endometriosis on gut microbiota (84). In a 2021 study conducted in Zhujiang, China, researchers found that patients with endometriosis have distinct microbial communities in their peritoneal fluid and feces compared to controls. In the peritoneal fluid of endometriosis patients, there were more pathogens, while there was a loss of protective microbes in the feces samples (34). Alterations to diet and supplementation may, therefore, be able to support improved microbial health, which may be able to support the reduction of endometriosis symptoms and even reduce the size of endometriotic lesions by impacting the microbiome (85). Of the 16% of participants that had chosen to alter their diet for endometriosis, half found it to be an effective treatment.

One participant shared her experience with the group of the lengthy recommendations from her endometriosis dietician. During a 4-month program, at a price of USD 250 a month, the dietician was able to identify foods that were triggers for endometriosis pain, and supplements that would help manage their symptoms. The foods the patient found they needed to remove to reduce their pain were caffeine, alcohol, garlic, onions, chili, dairy, gluten, corn, sugar, processed food, red meat, eggs, pork, peanuts, legumes, and brassicas. They also needed to increase their consumption of leafy greens, fiber, and fish, especially salmon for its rich source of omega-3 fatty acids. Some participants spoke very highly of the effect of diet changes on them, however, one patient (Confirmed, 25–30, Nulliparous) highlighted that: “when I found out that caffeine, alcohol, garlic, onions, chili and dairy all negatively impact me, I was not happy.” This quote highlights that while changes to diet can be effective for some patients, including half of the patients in this study that had attempted dietary changes for treatment, it can be perceived as yet another sacrifice the patient has to make because of their condition.

##### Acupuncture

Acupuncture is considered an effective method for the treatment of chronic pain, and is a procedure in which fine needles are inserted into the skin at specific points (86). This procedure is thought to stimulate the central nervous system to release chemicals into the muscles and central nervous system. This alteration to the body's biochemistry is thought to be able to stimulate healing and promote both emotional and physical well-being (87). Within this cohort all statements about acupuncture were positive. Similar forms of stimulation to acupuncture include the application of heat, pressure, friction and cupping (87). Acupuncture was a method of treatment 6% of patients wished they had started straight away as a method of pain management, and immensely positive statements were relayed:

“Acupuncture really helps me. I could walk in doubled over and leave being able to walk normally again, it's good to have a drug free option. This option is not offered by traditional medicine.” (Confirmed, 31–35, Parous)

#### Fertility treatments

Within this cohort, 12% of patients reported experiencing infertility that they considered to be related to their diagnosis of endometriosis. Amongst the patients of the cohort, 10% utilized fertility treatments, with 20% finding those treatments effective. Fertility issues are common amongst endometriosis patients, with up to 50% experiencing sub-fertility or infertility (88). Endometriosis-related infertility appears to result from a diminished ovarian reserve, compromised receptivity of the endometrium, disruption to the body's balance of estrogen and progesterone (88) or disrupted oocyte pick-up as a result of anatomical distortions (89). Infertility was not only a lived experience for 12% of the patients in this cohort but also a key source of fear and anxiety for another 10% of participants. For these patients, even though they had not yet attempted to become pregnant, knowing that a condition they could never fully treat could also eliminate or severely hinder their capacity to become and remain pregnant was a major concern. Patients frequently became aware of the impacts of endometriosis on fertility early in their endometriosis journey, either by being informed by their clinician or through online searches and tended to find the information overwhelming, as it cast further uncertainty over their futures. Many patients in the study shared their painful experiences of long periods of trying to conceive, multiple miscarriages, and the joy of their “rainbow babies”:

“I would say I was most devastated when I was googling my symptoms and endometriosis came up, especially as everywhere it was said that it affects a person's fertility. I badly wanted to become a Mum, to have children. Living with the fear of not being able to do this due to endometriosis was really really scary—along with the fact that there is no real cure to endometriosis.” (Confirmed, 25–30, Parous)

“I have unfortunately needed 4 rounds of IVF to conceive our first child and it was such a lot to go through. I am so glad I did it though!…The waitlist for [publicly] funded IVF was 18 months but think it could be getting longer with COVID and more people needing help. If I am honest, going private while expensive works better because the doctors do not hold back medication.” (Confirmed, 31–35, Parous)

## Discussion

### Recruitment limitations

Since the recruitment method employed for this study required an expression of interest from self-selected patients, this may have resulted in an over-representation of patients who are dissatisfied with their care, and therefore have a greater desire to share negative experiences, which could bias the results of this study toward representation of these experiences. In a 2007 study by Agarwal et al. about the motivations for endometriosis patients to participate in clinical and basic science studies, they found that the strongest motivating factors for participation were the “potential benefit to other women's health” followed by “dissatisfaction with current treatment options” and a desire to “improve [their] own condition” (90). In this study, over half of the patients highlighted that a reason for them wanting to participate was to improve the overall understanding of endometriosis, and 32% specifically highlighted they hoped sharing their stories would be beneficial to other endometriosis patients.

A second limitation of the study is that information about the ethnicities of study participants was not collected, however, some patients did identify this information in their answers. In New Zealand, 17.1% of the population is Māori (91), however, zero participants in our study self-identified as Māori. Compared to non-Māori, Māori patients in general face delayed treatment (92), lower life expectancies (93) and worse health outcomes (94, 95) in the New Zealand health system. Currently, there are no published studies that assess the perceptions and experiences of diagnosis and care of Māori cohorts experiencing endometriosis (mate kirikopu). In a 2022 study, 12.1% of the 620 patients identified as Māori (24) but the data was not separated for analysis from the overall cohort. Internationally, a trend has been observed of longer diagnostic delays for ethnic minorities (96) but it is not yet known whether this occurs in New Zealand. Future work to comprehend the differences in care for Māori endometriosis patients will be vital in determining any differences or disparities in their care and if their research priorities differ to those of non-Māori patients.

### Findings and recommendations

This cohort study has highlighted some key patient experiences with diagnosis and treatment of endometriosis that require attention and improvement in the near future. We believe these changes have the capacity to improve the experiences endometriosis patients have with diagnosis and treatment immensely.

#### Transvaginal ultrasound

The use of transvaginal ultrasound in the care of endometriosis patients should be assessed and carefully considered. The pain relayed with the procedure discussed by patients in this cohort was extreme. It seems prudent that a transvaginal ultrasound should only ever be applied to endometriosis patients by practitioners aware and proficient in the techniques specific to the detection of endometriosis, to improve the chance of accurate detection, and to potentially reduce the pain associated with the procedure. This procedure should not take place if the patient is unwilling to have it completed, as occurred in the recount of one patient within the study. It may also be beneficial to offer patients the ability to insert the probe themselves, as was highlighted by one patient (31–35, Confirmed, Nulliparous): “the person conducting it asked me to insert the wand, which made me feel more in power of my situation and more human.”

#### Hysterectomy

The role of hysterectomy in endometriosis care, and the perspective of patients about the prospect of hysterectomy, or their experience with hysterectomy should continue to be assessed. To date, studies of the outcomes of hysterectomy have been favorable for selected patients (97), a trend indicated amongst this cohort as well. The disparity between the favorable view of patients, and the views expressed by some of their medical practitioners, should be subject to further work in the future. For some patients, there were immense barriers to obtaining a hysterectomy, and it is important to note that while hysterectomy was one of the treatments with the lowest use by the cohort, it was the treatment with the highest rating of effectiveness by those users. Patients who wish to access a hysterectomy should not be subjected to sexist and demeaning statements described by patients in this study.

#### Increased awareness of endometriosis

Increasing public awareness of endometriosis will be of benefit to patients. If the impact of endometriosis, particularly the effects on the capacity to complete study and work, are better understood by the general public, this may reduce the negative responses some endometriosis patients have faced from their employers and their peers.

#### Improved treatment and reduced delay to diagnosis

There were low overall reported efficacies for available treatment methods by the patients of this cohort. This indicates there is likely a strong desire amongst patients for novel treatments to improve their condition. For new or improved treatment options to become available, the funding available for endometriosis research and endometriosis care will need to increase. To effectively reduce the delay to diagnosis, the funding allotted to the training and provision of gynecological specialists and facilities will also need to increase to create a proficient and larger workforce to clear the waitlist and backlog of endometriosis patients requiring treatment. Ongoing attention should also be paid to the experiences of patients with working diagnoses and their capacity to access effective endometriosis-focused holistic pain management schemes.

## Conclusions

For endometriosis patients, the key diagnostic tools employed were symptom history to give a preliminary, “working diagnosis,” abdominal and transvaginal ultrasounds, and laparoscopy that could confirm the presence of endometriosis. While experiences with abdominal ultrasound ranged from neutral to positive, experiences with transvaginal ultrasound were predominantly negative. For a procedure that should not hurt, and reportedly may only cause some discomfort (39), multiple patients were left in pain for multiple days following the imaging, with some even crying in pain during the procedure, and others being unable to have the procedure completed at all.

Experiences with laparoscopy were primarily positive, with many endometriosis patients being left with palpable relief that endometriotic lesions had been identified as an explanation of their symptoms. The negative experiences with laparoscopy centered around insufficient post-operative care, the need for repetitive treatments, and in the cases where endometriosis was missed during an initial laparoscopy, leaving patients with all their lesions and no relief. The most common feeling endometriosis patients felt after obtaining their endometriosis diagnosis was relief (88%). This reaction is emblematic of the difficulty involved in the journey an endometriosis patient often must go through to obtain this diagnosis. There are few other diagnoses where the disease is both chronic and incurable, where so many patients' first reaction is relief that at least they were not making it up, as one patient (25–30, Confirmed, Nulliparous) explained her relief that: “the surgery was not for nothing, that I might feel a bit better and that I would not look like a hypochondriac anymore.”

Endometriosis patients in this study's cohort had attempted a wide range of treatment options to reduce their endometriosis symptoms of pain, including pain relief medications (96%), laparoscopy (84%), COCPs (80%), exercise (66%), changing their diet (16%) and hysterectomy (12%). For the portion of patients that had used a certain treatment, the most effective treatments were hysterectomy (100%), laparoscopy (67%), neuropathic pain relief (50%), IUDs (50%) and diet changes (50%); whereas, the least effective were COCPs (25%), POPs (24%), counseling (20%), and fertility treatments (20%). The treatment that the greatest number of patients wished they had done much earlier was laparoscopy, while the treatment most patients wished they had skipped entirely were COCPs.

Endometriosis is a condition predominantly characterized by chronic pelvic pain, as well as painful disruptions to experiences such as menstruation, sex, ovulation, urination, defecation, and attempts to operate effectively in the workplace, in education, and in everyday life and relationships. The immense impact of this pain on the experiences of this cohort was evident, with 8% not working because of their disease and frequent reports of disruptions in school and the workplace. The intangibility of endometriosis for most patients, as scanning technology frequently cannot image the source of their symptoms, makes proving their pain to external parties challenging, and frequently leads to dismissal and minimization by others, including the clinicians that are supposedly in a position to help them.

## Data availability statement

The raw data supporting the conclusions of this article will be made available by the authors, without undue reservation.

## Ethics statement

The studies involving human participants were reviewed and approved by University of Canterbury Human Research Ethics Committee. The patients/participants provided their written informed consent to participate in this study.

## Author contributions

KE, DM, and RW contributed to the design of the questionnaire, KE recruited the participants, moderated the discussions, analyzed the results, and was the primary author of this paper through conception and analysis. DM and RW have contributed equally to this work through critical revision and editing. All authors contributed to this article and approved the submitted version.

## Funding

The Biomolecular Interaction Center provided seed funding to the Engineering Endometriosis Research Program (KE, DM, and RW) at the University of Canterbury in 2021. A portion of this funding was used to provide the study participants with inducements as an appreciation of their time.

## Conflict of interest

The authors declare that the research was conducted in the absence of any commercial or financial relationships that could be construed as a potential conflict of interest.

## Publisher's note

All claims expressed in this article are solely those of the authors and do not necessarily represent those of their affiliated organizations, or those of the publisher, the editors and the reviewers. Any product that may be evaluated in this article, or claim that may be made by its manufacturer, is not guaranteed or endorsed by the publisher.
